# Beneficial Effects of Oral Lactobacillus on Pain Severity in Women Suffering from Endometriosis: A Pilot Placebo-Controlled Randomized Clinical Trial 

**DOI:** 10.22074/ijfs.2019.5584

**Published:** 2019-07-14

**Authors:** Sepideh Khodaverdi, Robabeh Mohammadbeigi, Mojdeh Khaledi, Leila Mesdaghinia, Fatemeh Sharifzadeh, Somayyeh Nasiripour, Mansoureh Gorginzadeh

**Affiliations:** 1 Fellowship in Minimally Invasive Gynecologic Surgery (FMIG), Endometriosis Research Center, Iran University of Medical Sciences (IUMS), Tehran, Iran; 2 Fellowship in Infertility, Endometriosis Research Center, Iran University of Medical Sciences (IUMS), Tehran, Iran; 3 Endometriosis Research Center, Iran University of Medical Sciences (IUMS), Tehran, Iran; 4 Rasoul Akram Hospital, Iran University of Medical Sciences (IUMS), Tehran, Iran

**Keywords:** Chronic Pelvic Pain, Dysmenorrhea, Dyspareunia, Endometriosis, Lactobacillus

## Abstract

**Background:**

This study assessed the effects of a lactobacillus-based medication on pain intensity scores in women with endometriosis.

**Materials and Methods:**

The present randomized pilot placebo-controlled trial was done on eligible women who were surgically and pathologically diagnosed with endometriosis. Thirty-seven participants who had not received hormonal treatment in the last three months, were enrolled and randomized into LactoFem^®^and placebo groups. Lactobacillus capsules or placebo were administrated orally once a day for 8 weeks. Patients were assessed for pain severity using Visual Analogue Scale (VAS) scores for dysmenorrhea, dyspareunia and chronic pelvic pain at baseline and after 8 and 12 weeks post-intervention.

**Results:**

Mean age of participants and mean body mass index (BMI) for the LactoFem^®^and control groups were compara- ble. All patients had stage 3 and 4 of the disease based on revised American fertility society (AFS) classification of endome- triosis. Mean initial pain scores for dysmenorrhea, dyspareunia and chronic pelvic pain were 6.53 ± 2.88, 4.82 ± 3.76 and 4.19 ± 3.53, respectively in the LactoFem^®^group and 5.60 ± 2.06, 3.67 ± 2.64 and 2.88 ± 2.80, respectively for the control group; the two groups had comparable scores in this regard. There was more decrease in pain scores for both dysmenorrhea and the overall pain after 8 weeks of treatment in LactoFem^®^group compared to the control group. The scores for dysmen- orrhea were 6.53 ± 2.88 and 5.60 ± 2.06 in the LactoFem^®^and control groups, respectively, before intervention but, after 8-week treatment, these values were 3.07 ± 2.49 and 4.47 ± 2.13 (P=0.018), respectively. The changes in overall pain score in the LactoFem^®^ and control group during this period were 7.33 ± 7.00 and 4.11 ± 1.68, respectively (P=0.017).

**Conclusion:**

This study showed some beneficial effects of lactobacillus administration on endometriosis-related pain (Registration number: IRCT20150819023684N5).

## Introduction

Endometriosis, characterized by abnormal presence of endometrial tissue outside the uterus, is a major cause of discomfort in women (1, 2). This disease which occurs primarily in women of reproductive ages, seems to be an estrogen-dependent phenomenon (1-3). Although clinical symptoms are not seen in all women, the impact of endometriosis on physical, psychological and social performance is obvious in many other women (4). Endometriosis-associated pain includes dysmenorrhea, dyspareunia, dyschezia and dysuria, as well as chronic pelvic pain. Endometriosis patients at some time points endure debilitating pain which is worse than the pain experienced by women suffering from cancer (2). Moreover, ovarian endometriosis may have clinical and paraclinical manifestations of ovarian carcinoma (5). The mainstay of treatment of endometriosis consists of surgery accompanied by ovarian suppressive therapy (6, 7). Full consultation with patients and use of various types of analgesics, oral contraceptive pills, progestins or gonadotropin-releasing hormone agonists (GnRHa) are often required (8-12). 

There is sufficient evidence showing the efficacy of progestins and GnRHa against endometriosis- associated pain (13, 14), however, their side effects and patient tolerance, particularly in the long term, should not be overlooked (10, 13, 14). Based on molecular studies, changes in the function of immunologic cells like monocytes, macrophages, natural killer cells (NK), cytotoxic T cells and B cells have been detected in the peritoneal fluid of women with endometriosis. This alteration of immunologic defense which is not capable of removing the ectopic endometrial cells, leads to implantation of endometriosis lesions. Furthermore, the paramount role of NK cells was highlighted in many studies (15-20). According to Oosterlynck et al. (17), decreased activity of NK cells is remarkably associated with the severity of endometriosis. Previous studies led to the hypothesis that lack of ectopic endometrial clearance by NK cells in the peritoneal fluid contributes to the development of the disease. Therefore, any agent that stimulates the immune cells or increases the cytotoxicity of NK cells could be beneficial in treatment of endometriosis (21-24). 

Sashihara et al. (21-23) showed that a kind of lactobacillus called Lactobacillus gasseri (OLL2809), which is of probiotic type, stimulates the production of interleukin 12 (IL-12) from murine spleen cells. IL-12, a cytokine secreted by antigen presenting cells, triggers the production of cytotoxic lymphocytes by activating NK cells and T cells (25, 26). Lactobacillus species including *Lactobacillus acidophilus, Lactobacillus plantarum, Lactobacillus fermentum* and *Lactobacillus* gasseri, constitute the predominant normal microbial flora of genitourinary and gastrointestinal (GI) tract of healthy individuals. The effectiveness of these probiotics in maintenance of the normal pH of vagina and prevention of genital infections has been well-studied (27). Host immunity modification and interference with colonization of external pathogens are considered their main mechanisms of action (28, 29). There is also evidence that alterations in the normal flora within the gastrointestinal (GI) tract caused by administration of probiotics, antibiotics or even transplantation of feces into the GI tract, could result in pain relief by affecting neurologic pathways (30). In this regard, some recent studies indicated the use of lactobacillus-mediated medications in the treatment of endometriosis- related lesions (31, 32). Considering the hypothesis that lactobacillus may have immunogenic properties, the present study was conducted to assess the efficacy of oral lactobacillus-based pills on pain relief in patients diagnosed with endometriosis. 

## Materials and Methods

This was a pilot randomized triple-blind placebo-controlled trial carried out in a referral center for endometriosis in a university-based hospital in Tehran, Iran from October 2016 to October 2017. Enrolled participants were women with endometriosis (diagnosed based on pathologic report) who had undergone laparoscopic surgery due to pain and were randomly allocated into one of the two groups at a 1:1 ratio. The study was approved by the Institutional Review Board of Iran University of Medical Sciences (IUMS) by the Ethical Committee number IR.IUMS.REC1395.9311290013. All participants were patients with stage 3 and 4 of endometriosis (according to the revised American fertility society (AFS) classification of endometriosis (33). Patients were between 18 to 45 years old with menstrual cycle ranging from 21 to 35 days, with initial overall pain score higher than 4 [based on the visual analogue scale (VAS) scoring system]. The overall pain score was defined as the sum of dysmenorrhea, dyspareunia, chronic pelvic VAS pain scores. A scale of 0 (without any pain) to 10 (most severe pain), by the use of a 10-cm ruler in the questionnaire filled by the physician at the initial visit and each follow up visit at 8 and 12 weeks post-treatment, was used in the VAS scoring. Patients had at least 3 months interval from surgery and in this period, they were not supposed to use hormonal treatment; also, the participants were asked not to take any pain-killer medications other than NSAIDs which have short-term effects and do not have interference with lactobacillus effects. Those with history of hormonal replacement after surgery, hepatic or renal disturbances, cancer, diarrhea after taking dairy products, or consuming any type of probiotic products were excluded. Written informed consent was obtained from all patients eligible for the trial. Data including demographic findings, medical history and medication use were recorded in questionnaires by a physician in the first visit and completed in the follow-up visits at 8 and 12 weeks post-treatment visits. The participants were asked to mention any kind of excessive GI upset, nausea, vomiting, or any other non-specific side effects.

### Treatment protocol

**Fig 1:**
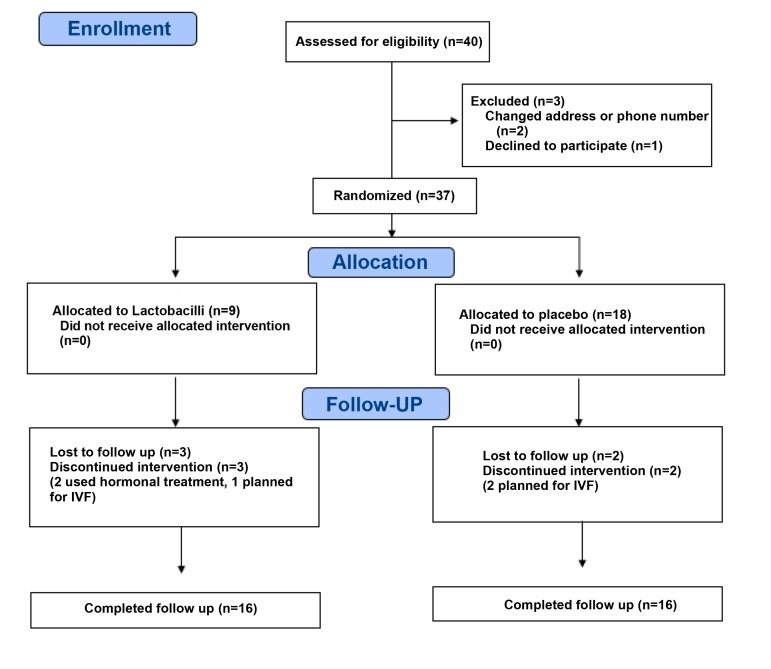
Flow diagram of the trial

The present study was a pilot placebo-controlled randomized clinical trial which recruited 20 patients for each arm (Fig.1). After exclusion of 3 patients, thirty-seven patients with endometriosis were randomly assigned (by simple randomization method using table of random numbers) to one of the two groups receiving either LactoFem^®^, Zist Takhmir Co. Tehran, Iran (one capsule per day) or placebo (as the control group). Each LactoFem^®^ capsule contains 10^9^ colony of four different lactobacillus strains (*Lactobacillus acidophilus, Lactobacillus plantarum, Lactobacillus fermentum* and *Lactobacillus gasseri*). The lactobacilli contents and placebo contents were packed in the similar packing with 30 capsules in each pack by the manufacturer; two packs were given to each patient in the first visit, to be used during the 8 weeks. The lactobacilli packs and placebo packs were named A or B by the manufacturer. After completion of the analysis, the manufacturer revealed which one was lactobacilli or placebo.

All women had undergone complete laparoscopic removal of endometriosis lesions including deep infiltrating endometriosis (DIE). The procedures had been performed with similar extent of resection including ovarian cystectomy (endometrioma), salpingectomy, ureteral dissection, uterosacral ligament ablation or DIE removal. The interval between surgery and commencement of intervention was at least 3 months. At the beginning of the study, patients were evaluated for the intensity of pelvic pain, dysmenorrhea, and dyspareunia based on the VAS score rated from zero (no pain) to 10 (the most severe pain). Patients in the two groups continued taking medication for 8 weeks and then, the pain intensity was evaluated again 8 and 12 weeks following intervention by a follow-up visit or a phone call. During the time of follow-up, patients were allowed to use NSAIDs only as the rescue therapy. Patients who were not willing to continue the trial due to personal reasons were excluded from the study. This study was conducted as a triple-blind trial in which the researcher, the subjects, and the statistician were all unaware of the allocation of the two groups. 

### Statistical analysis

Results are presented as mean ± SD for quantitative variables and as absolute frequencies and percentages for categorical variables. Normal distribution of data was assessed using the Kolmogorov-Smirnoff test. Categorical variables were compared using chi-square test or Fisher’s exact test. Quantitative variables were also compared using t test or Mann U test. ANOVA test was also used to analyze more than two means. For statistical analysis, the statistical software SPSS version 20 for windows (SPSS Inc., Chicago, IL) was used. P.0.05 were considered statistically significant. 

### Outcomes

The main outcome of the study was the mean pain score (for dysmenorrhea, dyspareunia and pelvic pain) after 8 and 12 weeks of intervention as assessed by VAS scoring system. The secondary outcome was the change in VAS scores during the first 8 weeks of intervention and from 8 to 12 weeks post medication.

## Results

The two groups were comparable regarding mean age (P=0.955), body mass index (BMI) (P=0.14), history of infertility (P=0.669), irregular menstrual cycle (P=0.264), underlying disorders (P=0.307), and history of medications (P=0.600). Demographic characteristics of the subjects are demonstrated in Table 1. All patients had undergone laparoscopy beforehand and endometriosis was pathologically diagnosed in all participants. According to revised American fertility society (AFS) classification of endometriosis (33), stage III was found in 25 and 0% and stage IV was observed in 75 and 100% of intervention and control groups, respectively (P=0.101). 

**Table 1:** Baseline characteristics of the participants


Parameter	Lactobacillus group	Control group	P value

Age (Y)	33.81 ± 6.85	33.69 ± 5.63	0.955
BMI (Kg/m^2^)	26.16 ± 5.46	23.64 ± 4.03	0.14
History of infertility	3 (18.8)	4 (25.0)	0.669
Irregular menses	7 (43.8)	4 (25.0)	0.264
Family history of endometriosis	3 (18.8)	1 (6.2)	0.600
Disease stage^*^			0.101
Stage III	4 (25.0)	0 (0.0)	
Stage IV	12 (75.0)	16 (100)	


Data are presented mean ± SD or n (%). BMI; Body mass index and *; Based on revised AFS classification.

As shown in Table 2, the mean pain scores at baseline as well as 8 and 12 weeks after intervention were not different between the groups. Using ANOVA analysis, the trend of the changes in pain intensity for dysmenorrhea, dyspareunia, and chronic pelvic pain during 12 weeks were evaluated. Concerning dysmenorrhea, the mean pain score decrease observed in the LactoFem^®^ group was significantly larger than that of the control group during 8 weeks of treatment (3.46 ± 2.97 vs. 2.18 ± 1.06, P=0.018). The decreases in mean pain scores from week 0 to 12 and from week 8 to 12 were not however statistically significant (P=0.051 and 0.191 respectively). Concerning chronic pelvic pain, the mean pain score decrease from week 0 to 8 was 3.35 ± 2.18 for the LactoFem^®^ group and 3.03 ± 0.37 for the placebo group (P=0.119). The decrease in chronic pelvic pain score from week 0 to 12 was not significant (P=0.458). The change in pain scores from week 8 to 12, however, was significantly larger in the control group (1.09 ± 1.00 vs. 1.34 ± 0.06, P=0.02). Concerning the overall pain scores, the mean pain score decreased significantly in the LactoFem^®^ group during 8 weeks of intervention in comparison to the placebo group (7.33 ± 7.00 vs. 4.11 ± 1.68, P=0.017). Moreover, the change in pain scores between week 8 and 12 was statistically different between the groups (P=0.015). No serious side effects following ingestion of these capsules were reported.

**Table 2:** Pain scores (VAS) at 3 different time points


Parameter	Lactobacillus group	Control group	P value

Dyspareunia			
Week 0	4.82 ± 3.76	3.67 ± 2.64	0.402
Week 8	2.55 ± 2.77	3.25 ± 2.30	0.513
Week 12	3.09 ± 2.59	3.17 ± 2.08	0.939
Change between week 0-8	-3.55 ± 2.27	-2.02 ± 0.38	0.117
Change between week 0-12	-2.86 ± 1.72	-2.96 ± 0.46	0.301
Change between week 8-12	0.93 ± 0.54	-1.97 ± 0.07	0.350
Dysmenorrhea			
Week 0	6.53 ± 2.88	5.60 ± 2.06	0.316
Week 8	3.07 ± 2.49	4.47 ± 2.13	0.110
Week 12	3.80 ± 2.54	4.60 ± 1.92	0.339
Change between week 0-8	-3.46 ± 2.97	-2.18 ± 1.06	0.018
Change between week 0-12	-2.73 ± 2.68	-1.66 ± 1.06	0.051
Change between week 8-12	1.75 ± 0.73	1.95 ± 0.00	0.339
Chronic pelvic pain			
Week 0	4.19 ± 3.53	2.88 ± 2.80	0.253
Week 8	2.00 ± 1.93	2.50 ± 2.34	0.515
Week 12	3.00 ± 2.39	2.44 ± 2.13	0.448
Change between week 0-8	-3.35 ± 2.18	-3.03 ± 0.37	0.119
Change between week 0-12	-3.22 ± 1.18	-2.33 ± 0.43	0.458
Change between week 8-12	1.09 ± 1.00	-1.34 ± 0.06	0.02
Overall pain score			
Change between week 0-8	-7.33 ± 7.00	-4.11 ± 1.68	0.017
Change between week 0-12	-6.86 ± 4.93	-4.05 ± 1.81	0.127
Change between week 8-12	2.47 ± 2.06	2.27 ± 0.12	0.015


Data are presented mean ± SD.

## Discussion

The aim of this study was to assess the therapeutic effects of oral lactobacillus on endometriosis-associated pain (including pain caused by dysmenorrhea, dyspareunia, and chronic pelvic pain). Few studies were conducted until now on the effects of lactobacilli on pain complaints related to endometriosis. A review of these few studies indicated the beneficial impact of lactobacilli on endometriosis (24, 31, 32). This possible effectiveness could result from increases in interleukin-12 levels and NK cells activity (15-18). Also, decrement of the activity of natural lethal cells seems to be related to the severity of endometriosis, and the inability to clear the ectopic endometrial lesions by the NK cells in the peritoneal space, contributes to development of disease (16-19, 22-24) which could be prevented by the use of probiotics. In a study done by Uchida and Kobayashi (32), lactobacillus therapeutic effect was evaluated in animal models following four weeks of treatment. It was finally observed that administration of lactobacillus was associated with a significant reduction in the volume of induced endometriosis in rats. 

In another study (31), 33 patients with clinical diagnosis of endometriosis were given Lactobacillus gasseri capsules for 12 weeks. It was shown that 2 and 3 months post- treatment, use of lactobacillus was associated with significant improvements in pain intensity during menstruation in comparison with placebo. This finding was consistent with ours. The difference in pain scores during the first 8 weeks were apparently more in the mentioned study (31), and this was due to the lower initial pain scores post-surgical treatment in the present study. In both studies, no significant relief in non-menstrual pain was achieved. In our study, diagnosis of endometriosis was based on pathologic report and not just based on complaints of dysmenorrhea or other types of pain, which could be a strength of the present study. Furthermore, surgical staging was done based on the revised AFS classification. All the subjects had gone through laparoscopic surgery because of intolerable pain. An interval of at least 3 months was given to each patient before prescribing lactobacillus, to evaluate the effects of the surgical treatment. Lactobacillus-based medication used in our study consisted of four different strains of Lactobacilli including *Lactobacillus gasseri* used by Itoh et al. (31). Although the mean pain scores for two groups (according to VAS) after 8 weeks and 12 weeks were comparable, a larger decrease in dysmenorrhea intensity and the overall pain scores in the LactoFem^®^ group was seen after 8 weeks of treatment. This improvement in pain after 8 weeks was not significant for chronic pelvic pain and dyspareunia comparing with dysmenorrhea and overall pain scores. Quite interestingly, during the four weeks following cessation of LactoFem^®^ (i.e. from week 8 to 12), the mean pain scores related to chronic pelvic pain and the overall pain intensity increased significantly compared to the control group. This increase could be due to the withdrawal effects of the LactoFem^®^ and the fact that the efficacy of the lactobacillus is limited to the treatment duration only. Our study was the first randomized trial using lactobacillus-based medication on stage 3 and 4 of endometriosis regarding three common pain types in such patients. Given the progressive nature of endometriosis and unbearable pain episodes related to this disorder, any intervention that could mitigate its symptoms, is certainly invaluable. Compared to other conventional medical therapies used for endometriosis-associated pain, LactoFem^®^ capsules have no remarkable side effects such as weight gain, flushing or abnormal uterine bleeding and no serious side effects following ingestion of these capsules were reported in our experiment.

Furthermore, these capsules modify microbiota of urogenital and GI tract and prevent from infections by improving immune system function. LactoFem^®^ capsules are readily available in our country at a reasonable price. The finding that the remedial outcome of LactoFem^®^ was not as significant as expected could be due to the limitations of our study. The first limitation was the small sample size which was not large as many patients had received hormonal therapy during 3 month interval before initiating the study. Also, some patients were not able to refer to the clinic for participation in the study. Another limitation that should be mentioned was the lower initial pain scores of the patients, due to the surgical treatment, which could affect both the sample size and the results. This trial was designed as a pilot study and we believe that in a larger study population, more robust results could be achieved. The dosage of lactobacillus capsules administered could be another limitation. Maybe at higher doses, more declines in pain scores could have been resulted. Moreover, changes in microbiome caused by lactobacilli were not evaluated which could be another limitation of this study. It should also be mentioned that it was not possible to design a cross-over study because of the limited time that many of the patients agreed to participate in the study, since many of them planned for *in vitro* fertilization (IVF) or pregnancy in the near future. Also, there was no similar study conducted within a longer time of follow-up to be sure how long the effect of lactobacilli could remain on pain suppression, therefore to avoid a bias in this field, we preferred a non-cross over design.

## Conclusion

It seems that lactobacilli have some beneficial effects regarding endometriosis-associated pain including dysmenorrhea and chronic pelvic pain. Regarding the dysmenorrhea, the best results happened after 8 weeks of the lactobacilli consumption, which also caused a significant decrease in the overall pain over the course of lactobacilli use in our study. The findings of our research may be used for sample size estimation for further randomized trials to better evaluate the impact of lactobacilli on endometriosis and its related symptoms.
